# Association between postoperative silver sulfadiazine dressings and surgical site infection after skin cancer excision: an observational cohort study with historical controls

**DOI:** 10.3389/fsurg.2026.1881730

**Published:** 2026-08-17

**Authors:** Barbara De Angelis, Davide Johan Bottini, Chiara Di Segni, Lorenzo Secondi, Marco Gerardi, Margarida Fernandes Lopes Morais D’Autilio, Nico Preite, Alfredo Pisapia, Pietro Gentile, Dario Crocoli, Daniele Di Giovanni, Mariachiara Carestia, Leonardo Palombi, Fabrizio Orlandi, Valerio Cervelli

**Affiliations:** 1 Department of Plastic and Reconstructive Surgery, University of Rome Tor Vergata, Rome, Italy; 2 PhD Program in Regenerative Surgery, University of Rome Tor Vergata, Rome, Italy; 3 Policlinico Casilino, Rome, Italy; 4 Plastic, Reconstructive and Aesthetic Surgery, University of Rome Tor Vergata, Rome, Italy; 5 Plastic and Reconstructive Surgery, University of Rome Tor Vergata, Rome, Italy; 6 Department of Biomedicine and Prevention, University of Rome Tor Vergata, Rome, Italy; 7 University “Our Lady of Good Counsel”, Tirana, Albania; 8 Division of Plastic and Reconstructive Surgery, University of Rome Tor Vergata, Rome, Italy

**Keywords:** basal cell carcinoma (basalioma), dermatologic surgery, observational cohort study, silver sulfadiazine, skin cancer, squamous cell carcinoma, surgical site infection (SSI), wound care

## Abstract

**Background:**

Surgical site infections (SSIs) remain a relevant postoperative complication in dermatologic surgery, negatively affecting wound healing, patient outcomes, and healthcare costs. Although the baseline infection risk after clean skin cancer surgery is relatively low, preventive strategies remain clinically important. Silver sulfadiazine (SSD) is a topical antimicrobial agent with broad-spectrum antibacterial activity; however, its preventive role in clean oncologic dermatologic surgery remains insufficiently investigated. This study evaluated the association between postoperative SSD-based dressings and SSI occurrence after skin cancer excision.

**Methods:**

A non-randomized observational cohort study with a non-concurrent historical control group was conducted including 800 patients undergoing excision of basal cell carcinoma or squamous cell carcinoma. The intervention group (*n* = 400) prospectively received postoperative SSD-based dressings, whereas the control group (*n* = 400) consisted of historical controls treated with paraffin gauze alone before implementation of the SSD protocol. The primary endpoint was SSI occurrence at postoperative day 3 (T1), defined as Southampton Wound Assessment Scale (SWAS) grade ≥ II. Relative risk (RR) and 95% confidence intervals (CIs) were calculated.

**Results:**

At T1, SSIs occurred in 36/400 patients (9.0%) in the SSD group compared with 72/400 patients (18.0%) in the control group (RR = 0.50; 95% CI: 0.34–0.73; *p* < 0.001). No additional infections were observed during subsequent follow-up evaluations (T2–T4). Recorded baseline demographic and clinical characteristics were broadly comparable between groups.

**Conclusion:**

Postoperative SSD-based dressings were associated with a lower incidence of early SSI after skin cancer excision. Because of the non-randomized design and the use of non-concurrent historical controls, the observed reduction should be interpreted as an association rather than proof of causality. Further prospective randomized studies are warranted.

## 1 Introduction

Surgical site infections (SSIs) represent the second most common healthcare-associated infection (HAI) after urinary tract infections. The 2011 WHO report on the burden of endemic healthcare-associated infection worldwide (1) indicated that SSIs were the most frequent type of HAI reported hospital-wide in low- and middle-income countries, where the level of risk is significantly higher than in high-income countries (2). SSIs are also the second most frequent type of HAI in the USA (3) and Europe (4). The global pooled incidence of SSIs was estimated to be 2.5% (95% CI: 1.6–3.7) in the 2023 systematic review and meta-analysis by Mengistu et al. (5), while the estimated prevalence ranges from 2% to 11% (6), despite numerous strategies implemented before, during, and after surgery to minimize infection risk.

The term SSI was coined by the United States Centers for Disease Control and Prevention (CDC) in 1992 (7). According to the CDC definition, SSIs are classified as superficial, deep, or organ/body cavity infections. Superficial SSIs develop within 1 month after surgery and involve the skin and subcutaneous tissue; deep SSIs develop within 1 month, or within 1 year if a foreign body has been implanted, and involve the fascia and muscles. Organ or body cavity infections may occur near the surgical site and develop within 1 month after surgery, or up to 1 year if a foreign body has been implanted. Surgical wounds are conventionally categorized by the CDC into four classes, each associated with a specific infection risk.

A clean wound has an infection risk of less than 2%; a clean-contaminated wound has an infection risk of 2%–10%; a contaminated wound has an infection risk of 10%–40%; and a dirty/infected wound has an infection risk of approximately 40%.

A range of factors affect the incidence of SSIs, including patient-specific variables such as comorbidities and procedural factors such as surgical technique and duration. Although patient-specific variables may be less likely than procedural factors to be the primary cause of SSI (8), the impact of SSIs extends beyond immediate clinical concerns, leading to prolonged hospital stays, increased healthcare costs, and substantial patient morbidity (9, 10). Recent studies have also focused on the skin microbiome as a potential risk factor for SSI development.

Gupta et al. (2023) investigated the microbiome of acute skin wounds after skin cancer surgery. The study included 65 patients and compared the microbiome of surgical wounds with that of intact skin using 16S rRNA sequencing. Key findings included a distinct microbial composition in wounds, with a significant reduction in *Cutibacterium*, which is common in normal skin, and an increase in *Staphylococcus aureus* and *Corynebacterium* species. The study suggested that *Corynebacterium* may play a role in preventing the pathogenicity of *S. aureus* in wounds (11).

Dermatologic surgical procedures are generally characterized by a high safety profile and a notably low incidence of complications. However, SSIs remain a clinically relevant complication, although infrequent. Prevention of these infections and their consequences can substantially affect patient outcomes, including wound healing, aesthetic results, and psychological well-being (12). These infections also have important implications for healthcare systems, influencing treatment costs and contributing to antimicrobial resistance (AMR) (8, 13). Reducing SSI rates is a common goal within the medical community because it may decrease costs, hospitalizations, and morbidity. Surgical site complications can also cause patient distress and create a financial burden due to prolonged or repeated wound treatment.

The escalation of antibiotic resistance presents a major challenge in surgical prophylaxis, particularly in plastic and reconstructive surgery. SSIs caused by antimicrobial-resistant pathogens are difficult to treat and may further complicate the clinical and economic impact of disease, although SSI rates and economic burden vary considerably across regions and countries (14). The evolving nature of antibiotic resistance requires careful reassessment of traditional prophylactic strategies for SSI prevention. Recent literature, including the analysis by Chiesa-Estomba et al. (2019), highlights the need to balance effective infection prevention with the risk of promoting resistant strains (15).

The role of antibiotic prophylaxis in preventing SSIs has shown mixed results. Chiesa-Estomba et al. suggested that antibiotic prophylaxis may not be essential in clean head and neck surgery. In contrast, Rosengren et al. (2018) demonstrated a significant reduction in SSIs with a single preoperative dose of antibiotics (2 g cephalexin) in complex dermatologic procedures on the nose and ear, particularly in male patients (16).

A scoping review covering evidence from 1945 to 2021 suggested that dressing characteristics may play an important role in outcomes after postoperative wound dressing. It also highlighted the need for dressing procedures to be aligned with antimicrobial stewardship principles to minimize the emergence of antimicrobial resistance (17).

SSI prevention is an essential goal in healthcare. It begins during the preoperative period, intensifies in the operating room, and continues throughout the postoperative phase.

During the preoperative phase, strategies to minimize SSI risk include smoking cessation for at least 4 weeks before and after surgery, assessment of nutritional status, including BMI and blood glucose level, management of immunosuppressive therapy, and appropriate use of antibiotic prophylaxis and anticoagulant therapy. Hair removal is another consideration, but it should be performed on the day of surgery by healthcare staff to avoid microtrauma that may promote SSI development.

The intraoperative phase carries the highest risk of infection. Preventive strategies include appropriate operating-room organization, separation of clean and contaminated pathways, air-quality control, maintenance of asepsis, surgical hand disinfection, and patient homeostasis (18–21).

Silver has broad antimicrobial activity against several bacteria, including multidrug-susceptible and resistant strains such as *Pseudomonas aeruginosa*, ESBL-producing *Escherichia coli*, methicillin-resistant *Staphylococcus aureus* (MRSA), and vancomycin-resistant *Staphylococcus aureus* (VRSA) (22). The antibacterial action of silver sulfadiazine is based on a dual mechanism: silver ions and sulfadiazine dissociate upon contact with exudate.

Sulfadiazine alters cellular membranes by inhibiting bacterial wall formation through interference with folic acid synthesis and facilitates the entry of silver ions into the cell. Inside the cell, silver ions react with DNA, blocking duplication and replication and disrupting bacterial DNA (23). Moreover, silver ions and silver nanoparticles exhibit toxicity to microorganisms by affecting respiratory enzymes and the electron transport system (24–28). Silver-based formulations, including silver sulfadiazine (SSD), have been reported to eradicate biofilms in burn wounds and chronic injuries, and may represent an alternative to traditional antimicrobials, particularly in topical applications at high concentrations (29). SSD has also been used to eradicate antibiotic-tolerant *Staphylococcus aureus* and *Pseudomonas aeruginosa* biofilms in patients with infected diabetic foot ulcers (30).

Van den Hoogenband conducted a study in patients with non-healing ulcers treated with partial-thickness skin grafts and reported that treatment with SSD cream before grafting improved healing. This improvement was attributed to a significant reduction in pathogens such as *S. aureus*, *Pseudomonas* spp., *Enterobacter*, and beta-hemolytic streptococci in the SSD-treated group (31).

In a study involving 71 patients with venous leg ulcers, Ouvry reported that SSD cream was effective in cleaning the ulcer and promoting subsequent granulation tissue formation (32).

Finally, the development of silver resistance in common opportunistic pathogens, including *Staphylococcus*, *Pseudomonas*, and *Enterobacter cloacae*, which are commonly found in chronic wounds, appears to be limited, although further investigation involving a broader range of strains and resistance mechanisms is needed (33).

However, most available evidence on silver sulfadiazine and silver-based formulations refers to burns, chronic wounds, infected wounds, or biofilm-related conditions. These data should not be directly extrapolated to clean surgical wounds after skin cancer excision, which represent a distinct clinical setting with different baseline infection risk and wound-healing dynamics. Therefore, the preventive role of SSD-based dressings in clean oncologic dermatologic surgery remains insufficiently established.

The present study is positioned at the intersection of surgical practice, infection control, and postoperative wound care. It aimed to evaluate the association between postoperative SSD-based dressings and early SSI occurrence after clean skin cancer excision.

Using an observational cohort design including 400 patients in the intervention group and 400 historical controls, the study sought to provide clinically relevant data on this postoperative dressing strategy in the context of skin cancer surgery.

This research is particularly relevant in the current context of antimicrobial resistance and the need for effective prophylactic approaches that are consistent with antimicrobial stewardship principles.

## 2 Materials and methods

### 2.1 Study design

This study was designed as a non-randomized observational cohort study with a non-concurrent historical control group.

### 2.2 Study population

A total of 800 patients (456 males and 344 females) undergoing surgical excision of skin cancer were included. Eligible patients were aged between 41 and 86 years and underwent excision of basal cell carcinoma or squamous cell carcinoma located on the head, neck, or body, with or without local flap reconstruction. None of the patients had undergone previous surgery in the same anatomical area.

Baseline characteristics were collected preoperatively and included age, sex, smoking status, comorbidities (diabetes mellitus, atrial fibrillation, arterial hypertension, chronic renal failure, and others), tumor type, and tumor site.

### 2.3 Inclusion and exclusion criteria

Inclusion criteria were:

- Age between 41 and 86 years;
- Diagnosis of basal cell carcinoma or squamous cell carcinoma;
- Surgical excision of skin cancer with primary closure or local reconstructive procedure;
- Availability for scheduled postoperative follow-up assessments.

Exclusion criteria were:

- Previous surgery in the same anatomical area;
- Previous local radiotherapy in the surgical area;
- Ongoing systemic chemotherapy or active oncological treatment at the time of surgery;
- Pre-existing local infection at the surgical site;
- Known allergy or hypersensitivity to silver sulfadiazine or sulfonamides;
- Inability to complete the scheduled postoperative follow-up.

### 2.4 Ethical considerations

The study was conducted in accordance with the Declaration of Helsinki and internationally accepted ethical standards.

The study protocol was approved by the Ethics Committee of the School of Medicine, University of Rome “Tor Vergata” (approval number: #27696), and by Rectoral Decree (D.R. n. 1693/2016, July 12, 2016).

All patients received detailed oral and written information and provided written informed consent prior to inclusion.

The treated group consisted of 400 patients who received postoperative silver sulfadiazine-based dressings (1% silver sulfadiazine cream and paraffin gauze), whereas the control group (*n* = 400) received standard paraffin gauze dressing.

### 2.5 Group definition and exposure

Patients were allocated to study groups according to the temporal implementation of the treatment protocol; no randomization was performed, and blinding was not feasible.

The intervention group consisted of 400 patients who prospectively received postoperative silver sulfadiazine-based dressings (1% silver sulfadiazine cream combined with paraffin gauze).

The control group (*n* = 400) consisted of a historical cohort of patients treated prior to the implementation of the silver sulfadiazine protocol, who received standard paraffin gauze dressings.

### 2.6 Methodological considerations

The use of a historical control group may introduce temporal and selection biases. In addition, treatment allocation was based on a predefined manual procedure managed by the clinical team, which may not fully eliminate allocation bias. These limitations were considered when interpreting the results.

Although the groups were broadly comparable with respect to the recorded baseline demographic and clinical variables, several procedural and perioperative factors potentially associated with SSI risk, including defect size, closure method, reconstructive complexity, operative time, anticoagulant use, glycemic control, immunosuppression, preoperative antibiotic use, and surgeon-related differences, were not consistently available in a standardized format for all patients. Residual confounding related to these unmeasured or incompletely recorded variables therefore cannot be excluded.

### 2.7 Clinical problem addressed

Baseline characteristics were collected preoperatively and included sex, age, smoking status, comorbidities (DM, diabetes mellitus; AF, atrial fibrillation; HD, hemodialysis; AH, arterial hypertension; and CRF, chronic renal failure), tumor type (basal cell carcinoma or squamous cell carcinoma), and tumor site.

Surgical wounds were assessed at predefined time points: T0 (immediate postoperative), T1 (3 days), T2 (6 days), T3 (time of suture removal), and T4 (30 days postoperative). Suture removal occurred at a mean of 9 days for head and neck procedures and 15 days for body procedures. The last evaluation was at T4 (30 days postoperative).

For the first three postoperative dressings (T0, T1, and T2), the intervention group received wound care consisting of irrigation with 0.9% NaCl, disinfection with sodium hypochlorite, application of 1% silver sulfadiazine cream, paraffin gauze, and a protective dressing. The control group received the same protocol without silver sulfadiazine.

Patients were instructed to keep the dressing clean, dry, and in place until the scheduled follow-up visits. Silver sulfadiazine was applied until T3 (suture removal), because this time point was considered to correspond to restoration of the skin barrier and reduced SSI risk.

Wound evaluation was performed using the Southampton Wound Assessment Scale (SWAS) (34), considering grades I–V as reported in Table 1. The primary endpoint was SSI occurrence at postoperative day 3 (T1), defined as SWAS grade ≥ II. Patients who met the criteria for SSI at T1 were counted as events in the primary outcome analysis. Thereafter, because the aim of the study was to evaluate SSI prevention rather than SSI treatment, patients with a positive SWAS score were managed according to clinical need and were not further evaluated as part of the standard preventive follow-up pathway. Additional postoperative complications, including hematoma, wound ischemia, wound dehiscence, or other events requiring alternative wound management, were recorded when present and considered in the clinical assessment.

**Table 1:** SWAS (Southampton Wound Assessment Scale) (34).

Grade	Definition
0	Normal healing
I	Normal healing with mild bruising or hematoma
II	Erythema plus other signs of inflammation
III	Clear or haemoserous discharge
IV	Pus
V	Deep or severe wound infection with or without tissue breakdown; hematoma requiring aspiration

### 2.8 Statistical analysis

All statistical analyses were performed using IBM SPSS Statistics, version 27.0.1.0 (IBM Corp., Armonk, NY, USA). Statistical significance was set at *p* < 0.05 (two-tailed). Continuous variables were summarized as mean ± standard deviation and categorical variables as frequencies and percentages. Baseline comparability between groups was assessed using Student's *t*-test for continuous variables and *χ*^2^ or Fisher's exact test for categorical variables, as appropriate. Standardized mean differences were also calculated.

The primary endpoint was postoperative wound infection at T1, defined as Southampton Wound Assessment Scale grade ≥ II.

This endpoint reflects early clinical inflammatory or infective wound changes and is not fully equivalent to CDC-defined surgical site infection. Infection rates were calculated for each group and reported with 95% confidence intervals. The unadjusted association between treatment group and infection at T1 was assessed using the *χ*^2^ test. Effect size was expressed as relative risk, absolute risk reduction, and number needed to treat. Secondary outcomes included SSI incidence at subsequent follow-up visits (T2–T4), wound-healing outcomes, and adverse events. Analyses beyond T1 were considered exploratory.

To account for potential confounding, a multivariable logistic regression model was fitted with infection at T1 as the dependent variable and treatment group as the main independent variable. The model was adjusted for age, sex, smoking status, number of comorbidities, tumour histology, and anatomical tumour region. Adjusted odds ratios with 95% confidence intervals were reported.

Given the observational design and the use of historical controls, results should be interpreted with caution because of the potential for residual confounding and temporal bias.

No formal *a priori* sample size calculation was performed. The sample size was determined by the number of consecutive eligible patients treated during the study periods.

## 3 Results

### 3.1 Study population

A total of 800 consecutive patients were included in the study, comprising 400 patients who received postoperative silver sulfadiazine (SSD)-based dressings and 400 historical controls treated with standard paraffin gauze dressings.

Baseline demographic and clinical characteristics are presented in Table 2. The two groups were comparable with respect to age, sex, smoking status, number of comorbidities, tumour histology and anatomical tumour region. No statistically significant baseline differences were observed, and standardized mean differences indicated a satisfactory balance between groups.

**Table 2:** Baseline characteristics.

Variable	SSD group (*n* = 400)	Control group (*n* = 400)	*p*-value	SMD
Age, years	63.59 ± 12.99	63.16 ± 13.06	0.635	0.034
Sex			0.721	0.030
Female	171 (42.8%)	177 (44.2%)		
Male	229 (57.2%)	223 (55.8%)		
Smoking status			0.855	0.039
Never smoker	236 (59.0%)	230 (57.5%)		
Current smoker	104 (26.0%)	111 (27.8%)		
Former smoker	60 (15.0%)	59 (14.8%)		
Number of comorbidities			0.170	0.155
None	170 (42.5%)	160 (40.0%)		
One	88 (22.0%)	115 (28.7%)		
Two	126 (31.5%)	112 (28.0%)		
Three	16 (4.0%)	13 (3.2%)		
Histology			1.000	0.000
Basal cell carcinoma	235 (58.8%)	235 (58.8%)		
Squamous cell carcinoma	165 (41.2%)	165 (41.2%)		
Tumour anatomical region			1.000	0.000
Head and neck	263 (65.8%)	263 (65.8%)		
Trunk	68 (17.0%)	68 (17.0%)		
Upper limb	24 (6.0%)	24 (6.0%)		
Lower limb	45 (11.2%)	45 (11.2%)		

SMD, standardized mean difference.

### 3.2 Primary outcome

At the immediate postoperative assessment (T0), all surgical wounds showed normal healing [Southampton Wound Assessment Scale (SWAS) grade 0], with no evidence of skin compromise, external bleeding or haematoma.

The primary endpoint was evaluated at postoperative day 3 (T1). Surgical site infection (SSI), defined as SWAS grade ≥ II, occurred in 36 of 400 patients (9.0%) in the SSD group and in 72 of 400 patients (18.0%) in the historical control group.

This difference was statistically significant [*χ*^2^(1, *N* = 800) = 13.87, *p* < 0.001]. The unadjusted relative risk of SSI was 0.50 (95% CI 0.34–0.73), corresponding to an absolute risk reduction of 9.0% and a number needed to treat (NNT) of 11.

**Figure 1:**
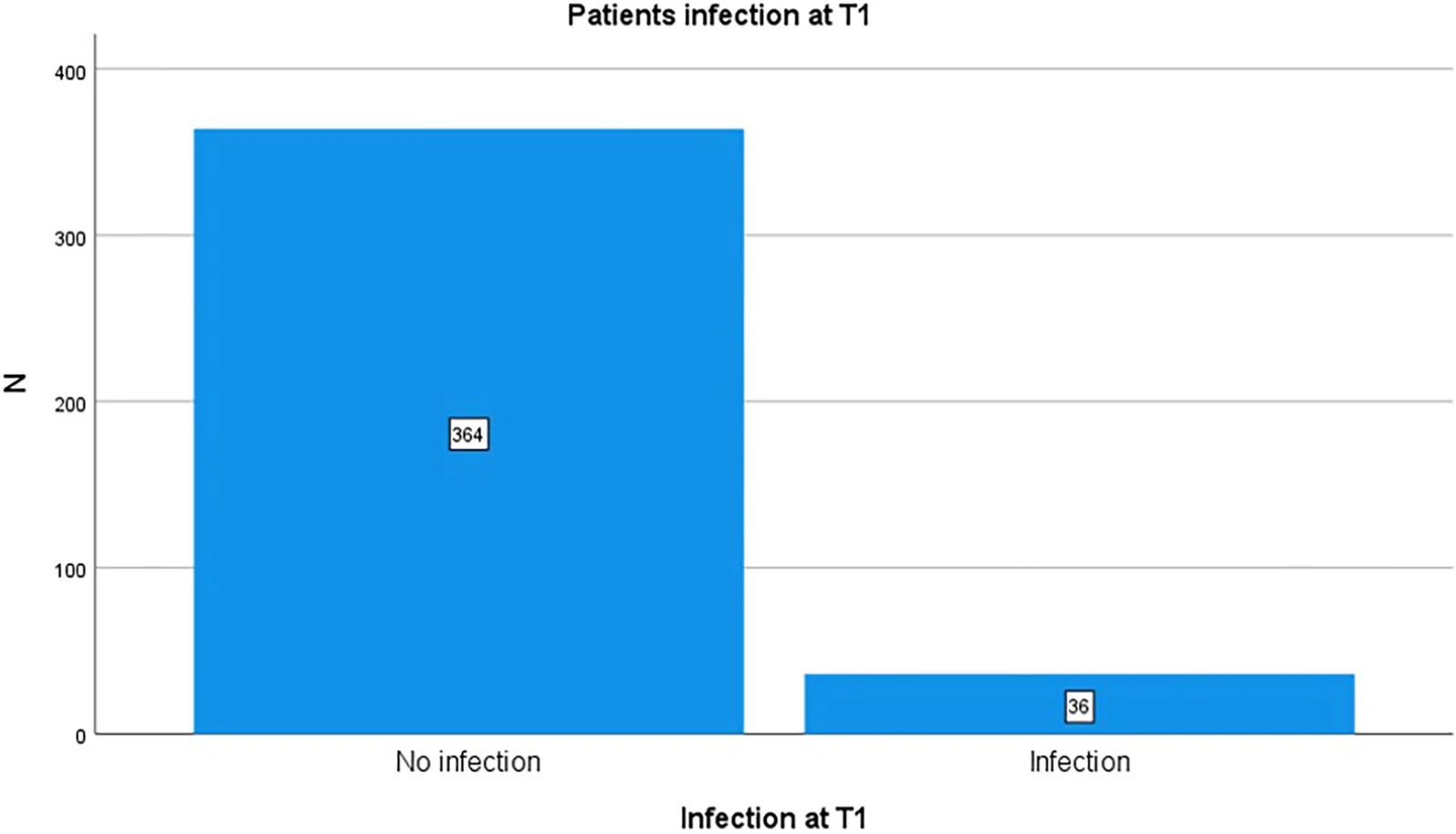
Incidence of SSIs at T1 in the SSD group.

**Figure 2:**
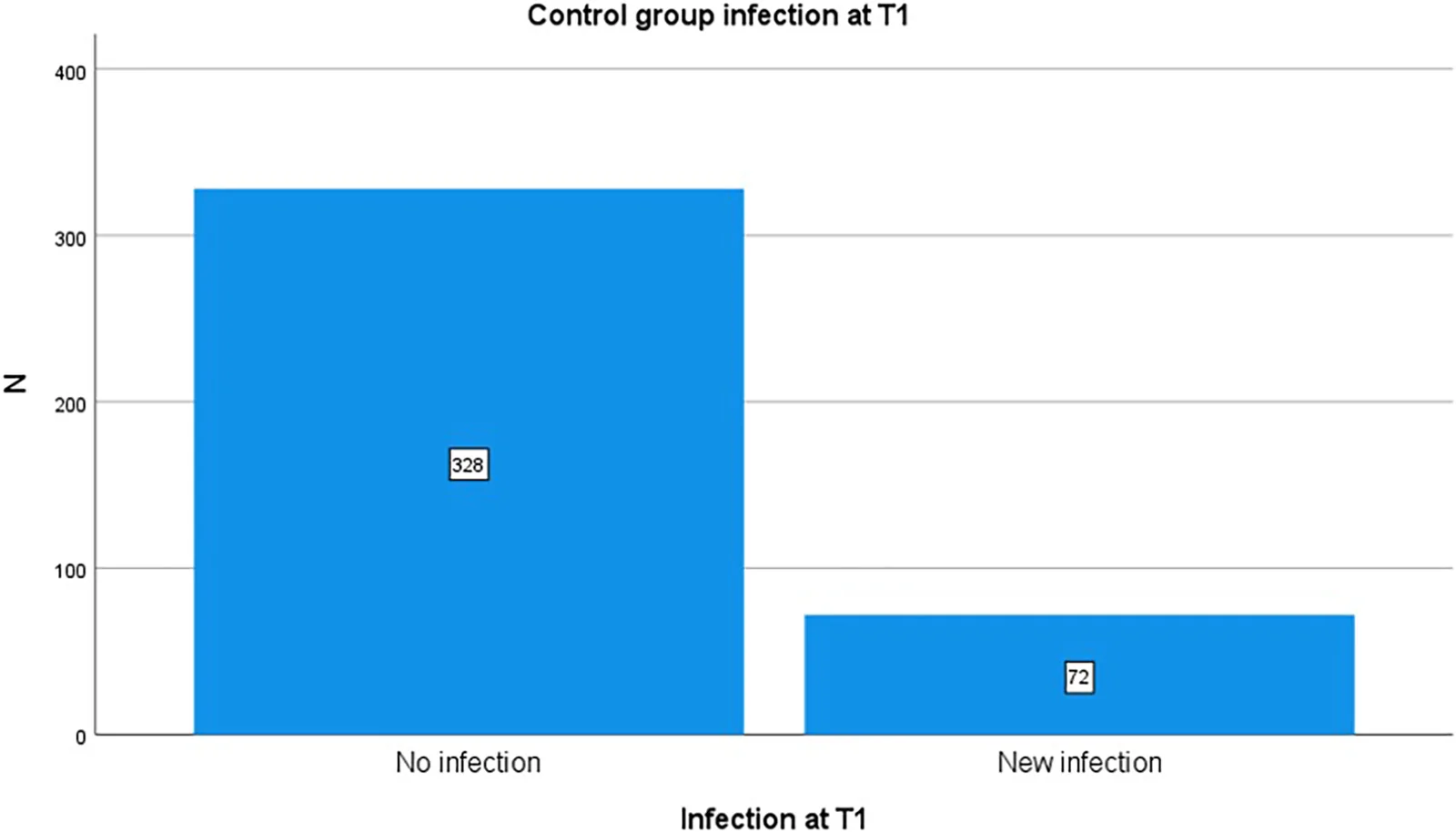
Incidence of SSIs at T1 in the control group.

The complete results of the primary analysis are summarized in Table 3. The distribution of infection status at T1 in the SSD and control groups is shown in Figures 1, 2, respectively.

**Table 3:** Primary outcome at T1.

Outcome	SSD group (*n* = 400)	Control group (*n* = 400)	Effect estimate
SSI at T1	36 (9.0%)	72 (18.0%)	
No SSI at T1	364 (91.0%)	328 (82.0%)	
*χ*^2^ test			*χ*^2^(1, *N* = 800) = 13.87; *p* < 0.001
Relative Risk			0.50 (95% CI 0.34–0.73)
Absolute Risk Reduction			9.0%
Number Needed to Treat			11

### 3.3 Adjusted analysis

To account for potential confounding related to the observational design and the use of historical controls, a multivariable logistic regression analysis was performed.

After adjustment for age, sex, smoking status, number of comorbidities, tumour histology and anatomical tumour region, postoperative SSD treatment remained independently associated with a significantly lower likelihood of SSI at T1 (adjusted OR = 0.41, 95% CI 0.26–0.64; *p* < 0.001).

Among the covariates included in the model, an increasing number of comorbidities was independently associated with a higher risk of SSI, whereas age, sex, tumour histology and anatomical region were not significantly associated with the outcome.

The complete regression model is reported in Table 4.

**Table 4:** Crude and adjusted logistic regression analysis of factors associated with SSI at T1.

Variable	Crude OR (95% CI)	*p*-value	Adjusted OR (95% CI)	*p*-value
Treatment group				
Control	Reference	—	Reference	—
SSD dressing	**0.45 (0.29–0.69)**	<0.001	**0.41** (**0.26–0.64)**	<0.001
Age, per year	1.01 (0.99–1.03)	0.214	1.01 (0.99–1.03)	0.197
Sex				
Female	Reference	—	Reference	—
Male	0.96 (0.64–1.44)	0.831	1.11 (0.72–1.72)	0.625
Smoking status				
Current smoker	Reference	—	Reference	—
Former smoker	0.58 (0.30–1.12)	0.107	0.66 (0.33–1.32)	0.236
Never smoker	0.57 (0.37–0.90)	0.014	0.61 (0.38–0.98)	0.039
Number of comorbidities				
None	Reference	—	Reference	—
One	3.06 (1.69–5.57)	<0.001	2.90 (1.57–5.34)	0.001
Two	3.71 (2.11–6.55)	<0.001	4.02 (2.25–7.18)	<0.001
Three or more	13.30 (5.59–31.62)	<0.001	14.57 (5.80–36.62)	<0.001
Histology				
Basal cell carcinoma	Reference	—	Reference	—
Squamous cell carcinoma	0.89 (0.59–1.35)	0.592	0.98 (0.63–1.52)	0.931
Tumour anatomical region				
Head and neck	Reference	—	Reference	—
Lower limb	1.07 (0.55–2.08)	0.836	1.35 (0.67–2.70)	0.400
Trunk	1.20 (0.70–2.06)	0.505	0.97 (0.55–1.73)	0.918
Upper limb	1.83 (0.87–3.86)	0.109	1.98 (0.90–4.40)	0.091

Bold values indicate statistically significant associations, defined as *p* < 0.05.

### 3.4 Follow-up

No additional cases of SSI were observed during the subsequent follow-up assessments (T2–T4). Since the primary endpoint had been prespecified at postoperative day 3, analyses performed at later follow-up visits should be considered exploratory.

**Figure 3:**
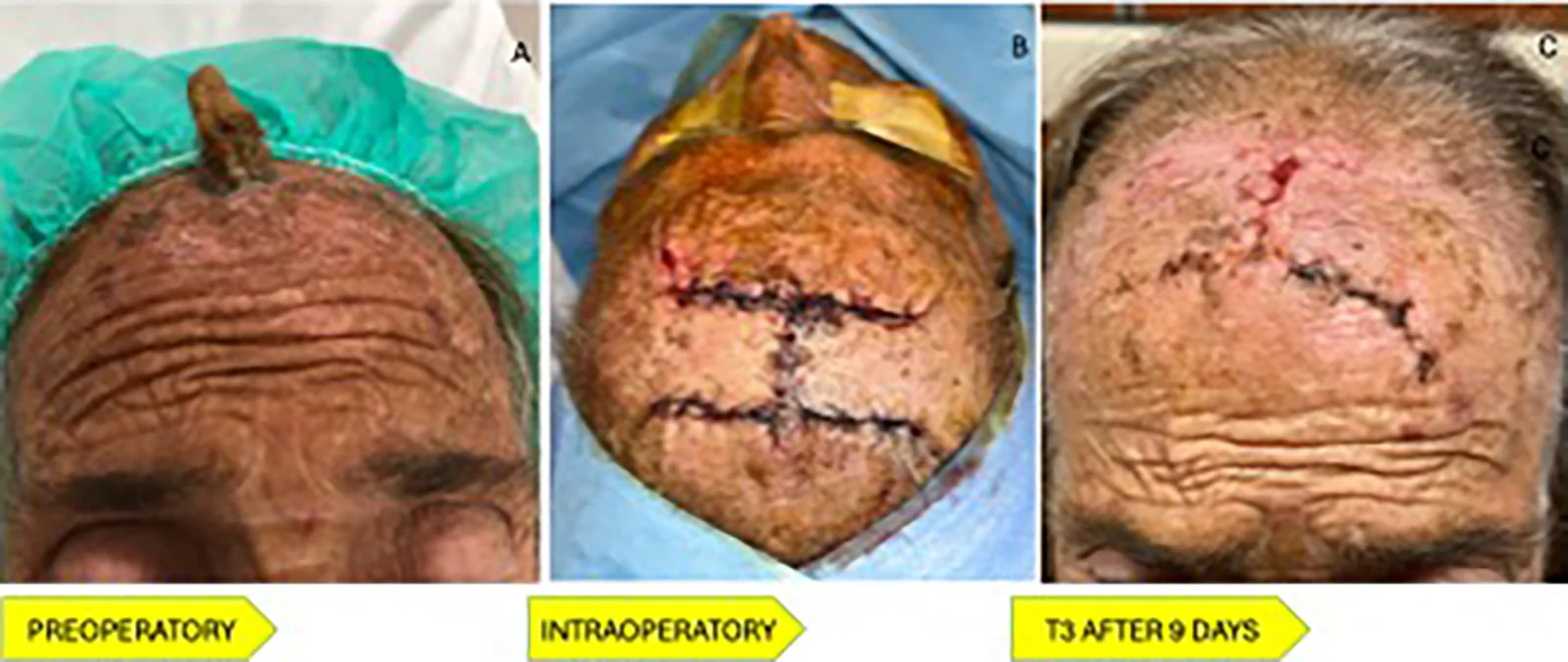
Patient with squamous cell epithelioma in the head region. **(A)** Preoperative view. **(B)** Intraoperative view after radical excision, reconstruction, and suturing without application of 1% silver sulfadiazine cream. **(C)** Result after 9 days, showing a complicated suture area.

**Figure 4:**
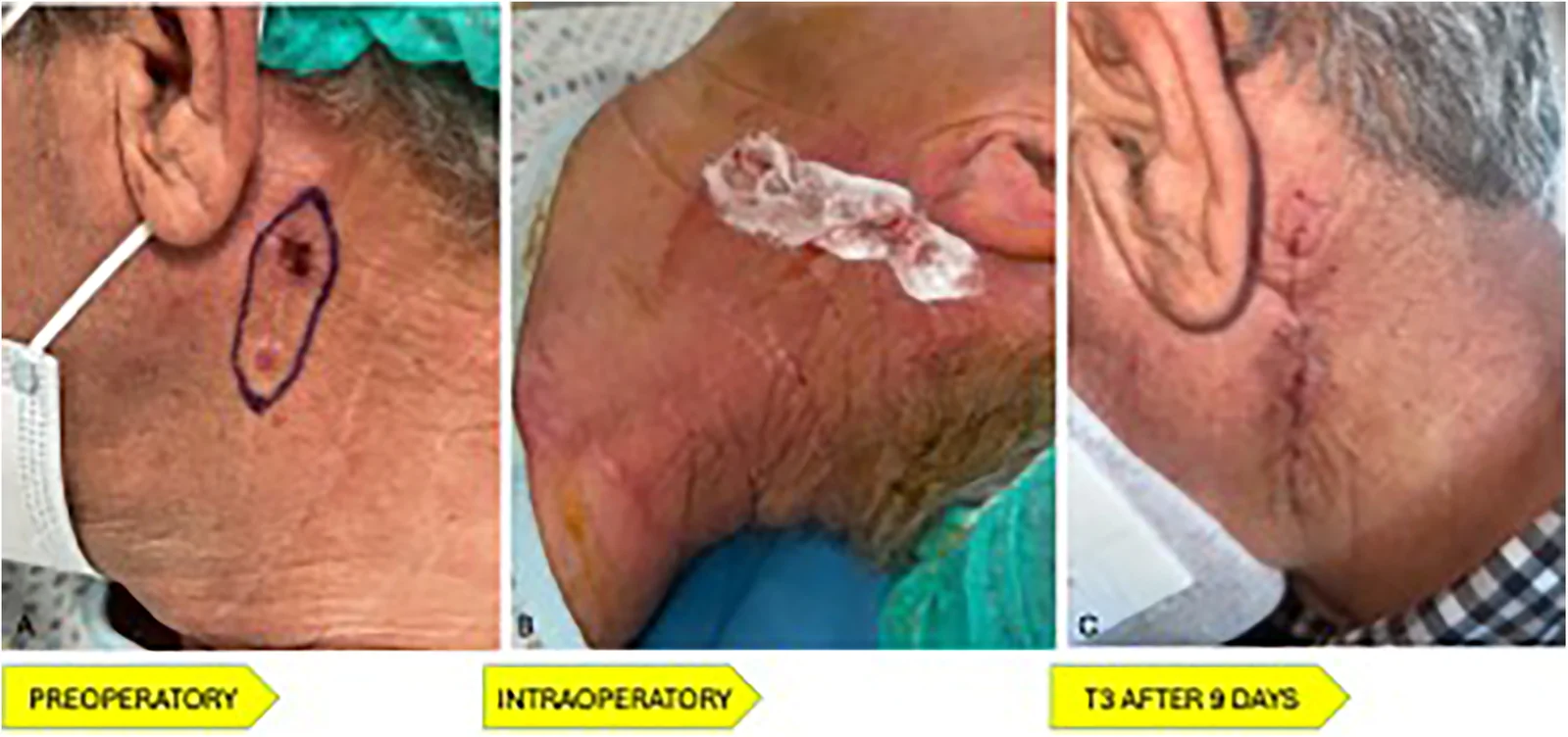
Patient with multiple basal cell epitheliomas in the neck region. **(A)** Preoperative view. **(B)** Intraoperative view after radical excision, reconstruction, and suturing with application of 1% silver sulfadiazine cream. **(C)** Result after 9 days.

Representative clinical cases illustrating postoperative wound healing in patients treated with standard dressings and SSD-based dressings are shown in Figures 3, 4.

Clinical examples of wounds treated with and without silver sulfadiazine are shown in Figures 3, 4.

Case 1: A patient with squamous cell epithelioma in the head region (Figure 3).
Case 2: A patient with multiple basal cell epitheliomas in the neck region (Figure 4).

## 4 Discussion

The human skin hosts a complex microbial ecosystem that acts both as a protective barrier and as a potential reservoir for opportunistic pathogens involved in postoperative wound colonization and SSI development (35). Its composition varies according to anatomical location, local humidity, sebaceous gland density, environmental exposure, hygiene, and host-related factors such as comorbidities (35, 36). Among the microorganisms commonly identified on human skin are species belonging to the genera *Staphylococcus*, *Corynebacterium*, *Cutibacterium*, *Streptococcus*, and *Pseudomonas*, several of which may contribute to SSI development under favorable conditions (36, 37).

In the context of SSI prevention, *Staphylococcus aureus*, including methicillin-resistant strains, remains one of the most relevant pathogens, and antimicrobial resistance continues to represent an important concern for postoperative wound management (38, 39). These considerations support the need for preventive strategies consistent with antimicrobial stewardship principles (40). However, in the present study, microbiological cultures were not systematically performed, and the observed reduction in early SSI was assessed clinically using the SWAS scale. Therefore, the microbiological rationale should be considered supportive rather than directly demonstrated by the present data.

In this cohort, postoperative SSD-based dressings were associated with a lower incidence of early SSI at T1 compared with paraffin gauze alone. This finding should be interpreted in light of the observational design, the use of a historical control group, and the SWAS-based endpoint definition. While the antimicrobial properties of SSD provide a plausible biological rationale, the present data do not allow definitive conclusions regarding causality or routine prophylactic use.

The chi-square test showed a statistically significant association between SSD-based dressings and reduced early SSI incidence compared with paraffin gauze alone. This finding is clinically relevant because even early postoperative wound complications may affect patient outcomes and healthcare resource use.

The observed association suggests that the dressing protocol may contribute to reducing early SSI occurrence in patients undergoing basal cell and squamous cell carcinoma excision. However, because of the observational design and historical control group, the result should be interpreted cautiously and does not establish causality. The resolution of infection, which is a prerequisite for subsequent wound healing, remains a primary objective of wound management (41).

The infection rate observed in the historical control group should not be interpreted as representative of all clean surgical wounds or of clean surgery in general. The present study focused specifically on patients undergoing skin cancer excision and reconstruction for basal cell carcinoma or squamous cell carcinoma, including elderly subjects, different anatomical sites, comorbidities, and variable reconstructive complexity. In addition, the primary endpoint was defined as SWAS grade ≥ II at postoperative day 3, which may capture early and mild postoperative inflammatory changes according to the predefined study definition. Therefore, the control-group infection rate should be interpreted within this specific clinical and methodological context.

This study has some limitations that should be acknowledged. First, no formal adjustment for multiple comparisons was applied across repeated time-point analyses (T1–T4), which may increase the risk of type I error. However, the primary endpoint was clearly pre-specified at T1, and analyses at subsequent time points were considered exploratory.

Second, the use of a historical control group may introduce temporal bias and limit comparability between groups, as differences in clinical practice, patient characteristics, or perioperative management over time cannot be completely excluded.

Despite these limitations, the relatively large sample size and the use of a clinically comparable historical control group support the relevance of the findings, while causal inference remains limited.

## Conclusion

Despite the implementation of infection control strategies and advances in surgical practice, surgical site infections remain a clinically relevant postoperative complication, with potential consequences for wound healing, patient outcomes, and healthcare resource utilization. Accurate assessment of patient-related risk factors, comorbidities, and perioperative variables remains essential, together with meticulous care during the preoperative, intraoperative, and postoperative phases.

In wounds healing by primary intention, evidence regarding the optimal postoperative dressing strategy for SSI prevention remains limited. The 2016 Cochrane review reported that topical antibiotics may reduce the relative risk of SSI compared with no topical antibiotic or topical antiseptics (42). However, uncertainty remains as to whether covering surgical wounds with dressings reduces SSI risk, and whether any specific dressing is superior in preventing infection, improving healing, reducing pain, enhancing patient acceptance, or facilitating dressing removal.

In the present observational cohort study with historical controls, postoperative dressings including 1% silver sulfadiazine were associated with a lower incidence of early SSI after skin cancer excision compared with paraffin gauze alone. Importantly, clean oncologic dermatologic surgery represents a specific clinical setting, and evidence derived from burns, chronic wounds, infected wounds, or biofilm-related conditions should not be directly extrapolated to clean surgical wounds after skin tumor excision. Therefore, the present findings should be interpreted within the specific context of clean skin cancer surgery.

Because of the non-randomized design, the use of a non-concurrent historical control group, and the possibility of residual confounding and temporal bias, the observed reduction in early SSI should be interpreted as an association rather than proof of a causal effect. No additional infections were observed during subsequent follow-up evaluations. During routine postoperative assessment, no obvious safety signals requiring discontinuation of the dressing protocol were recorded. Nevertheless, adverse events such as delayed wound healing, local irritation, allergic reactions, or silver-related toxicity were not predefined as formal study endpoints and were not systematically assessed. Similarly, no formal cost or cost-effectiveness analysis was performed. Therefore, conclusions regarding safety, tolerability, low cost, or economic advantage cannot be drawn from the present data.

Overall, SSD-based postoperative dressings may represent a potentially useful strategy for reducing early SSI occurrence after skin cancer excision. Further prospective randomized studies are warranted to confirm these findings and should include systematic assessment of adverse events, wound-healing outcomes, patient-reported outcomes, and economic endpoints before any definitive recommendation can be made for routine use in clean oncologic dermatologic surgery**.**

## Data Availability

The raw data supporting the conclusions of this article will be made available by the authors, without undue reservation.
